# Andrographolide is an Alternative Treatment to Overcome Resistance in ER-Positive Breast Cancer via Cholesterol Biosynthesis Pathway

**DOI:** 10.21315/mjms2019.26.5.2

**Published:** 2019-11-04

**Authors:** Harishini Rajaratinam, Siti Norasikin Mohd Nafi

**Affiliations:** 1 Department of Pathology, School of Medical Sciences, Universiti Sains Malaysia, Kota Bharu, Kelantan, Malaysia; 2 Department of Pathology, Hospital Universiti Sains Malaysia, Kota Bharu, Kelantan, Malaysia

**Keywords:** andrographis, ER-positive breast cancer, treatment resistance, cholesterol biosynthesis

## Abstract

Oestrogen receptor (ER)-positive breast cancer is one of the common forms of breast cancer affecting women worldwide. ER-positive breast cancer patients are subjected to anti-oestrogen therapy such as selective oestrogen receptor modulator (SERM) and aromatase inhibitors (AIs). Recently, the emergence of resistance to anti-oestrogen treatment is under intensive focus. The different mechanisms postulated to explain the occurrence of resistance in ER-positive breast cancer treatment include the loss of ER function and the crosstalk between signalling pathways in cancer cells. Recent literature highlighted that the cholesterol biosynthesis pathway acts as a novel mechanism underlying resistance to oestrogen deprivation. The present study aimed to highlight the role of cholesterol biosynthesis in anti-oestrogen treatment resistance, putatively suggesting an alternative plant-based treatment using andrographolide from *Andrographis paniculata*. The hypolipidaemic effect of andrographolide can be utilised to prevent the resistance in the treatment of ER-positive breast cancer contributed by cholesterol biosynthesis.

## Introduction

**Figure 1:**
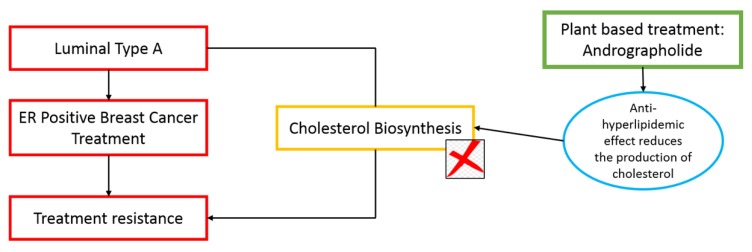
Illustration describing the andrographolide-mediated inhibition of resistance in ER-positive breast cancer due to cholesterol biosynthesis

Oestrogen-receptor (ER)-positive breast cancer can be treated with various forms of anti-hormone treatment. The anti-hormone treatments are commonly used to treat hormone-dependent cancer cells by inhibiting the supply of hormones required to promote the proliferation of these cells. Subsequently, the anti-hormone treatment might cause resistance to the treatment (1). Recent studies found that 25-hydroxycholesterol molecules were produced from cholesterol biosynthesis pathway might promote the resistance to the treatment involving oestrogen deprivation by mimicking the hormone (2). Based on this review, we analysed the potential of andrographolide, the bioactive compound of *Andrographis paniculata* in reducing the production of cholesterol that is required for the growth and resistance of cancer cells. In this review, we promoted the use of a natural product as an alternative treatment to replace conventional breast cancer treatment. Theoretically, the plant-derived anti-cancer drugs are considered more effective and safer for patients without significant side effects as compared to synthetic drugs. Natural products can target multiple vulnerabilities of the cancer cells, which aids in the prevention of reoccurrence and retardation of the disease (3). The hypolipidemic effects of andrographolide have been highlighted in various studies (4–5). The ability of andrographolide to reduce cholesterol production in cancer cells allows the incorporation of the bioactive compound in the treatment of ER-positive breast cancer. Cholesterol biosynthesis occurs via the mevalonate pathway, which consists of various enzymes and precursors. 3-Hydroxy-3-Methylglutaryl-CoA Reductase (HMGCR), is one of the rate-limiting enzymes found within the mevalonate pathway that produces cholesterol and steroid hormones by converting 3-hydroxy-3-methylglutaryl-CoA (HMG-CoA) (6). In a recent study, we found that andrographolide inhibits the proliferation of ER-positive breast cancer by downregulating the expression of HMGCR (unpublished data). Therefore, we provided adequate evidence supporting the hypothesis that andrographolide prevents the occurrence of resistance among ER-positive breast cancer patients via the inhibition of the cholesterol biosynthesis and the production of 25-hydroxycholesterol molecules (Figure 1).

### Luminal Type A: ER-Positive Breast Cancer

ER and progesterone receptors (PR) are present on hormone-dependent breast cancer cells such as luminal type A breast cancer cells. The proliferation of such breast cancer cells depends on the availability of steroid hormones such as oestrogen and progesterone. These receptors receive signals from the oestrogen and progesterone molecules, respectively, in order to promote the growth of the cancerous cells. Approximately, 30%–70% of the breast cancers are luminal type A tumours (7).

Luminal type A breast tumours have low histological grade, low degree of nuclear pleomorphism and low level of mitotic activity with good prognosis. It includes distinct histological types such as tubular, invasive cribriform, mucinous and lobular. Luminal type A is characterised by high levels of ER and low levels of proliferation-related genes (8).

**Figure 2:**
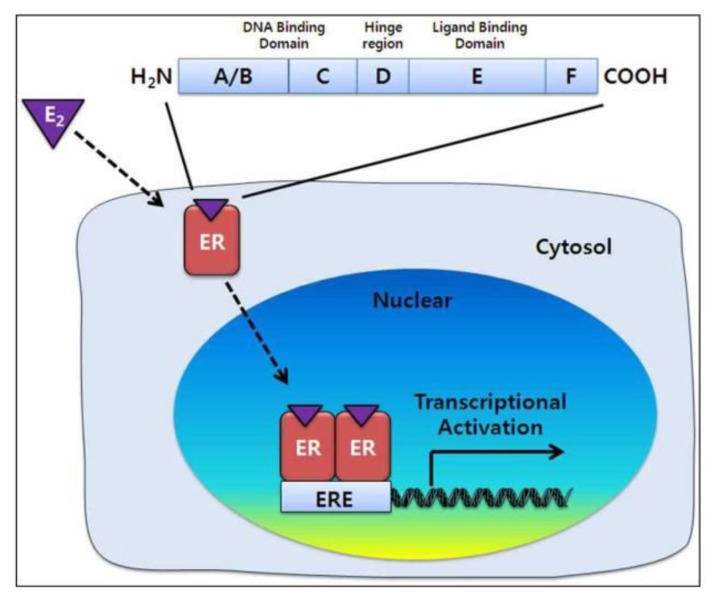
Mechanism of oestrogen receptor signalling pathway [figure taken from Lee et al. (9)]

Oestrogen signalling pathways are selectively regulated and dependent on the balance of the activities of the ER isoforms, ERα or ERβ, in target organs. The mechanism of oestrogen receptor signalling pathway involves a series of steps. Oestrogen molecules bind to ERs, which lead to the dimerisation and translocation of ERs. Then, these dimerised forms of ERs translocate to the nucleus and bind to the oestrogen response elements (EREs). ERs contain five domains with unique functions. The A/B domain contains the transcriptional activation function 1 (AF1) region, whereas the C domain contains the DNA binding domain. The D domain is a hinge region, and the E/F domain encodes the transcriptional activation function 2 (AF2) region (9) (Figure 2).

### ER-Positive Breast Cancer Treatment

While combating ER-positive breast cancer, physicians across the world increased the use of hormone therapy and chemotherapy. Among the types of hormone therapies, luteinising hormone-releasing hormone agents (LHRHs), aromatase inhibitors (AI), selective oestrogen-receptor response modulators (SERMs) and oestrogen-receptor down-regulators (ERDs) (10), the most preferred type of treatment in treating ER-positive breast cancer patients is SERMs. The molecular form of SERM lacks the steroid structure resembling that of oestrogen. However, the presence of a tertiary structure in its molecular arrangement allows SERM to bind to the ERs (11), usually throughout the body and act as tissue-specific oestrogen agonists or antagonists. Moreover, they prevent the growth of breast cancer cells by replacing oestrogen in the receptors to prevent detrimental effects. Tamoxifen is the most commonly prescribed type of SERM. It manipulates the influence of ER on the utilisation of oestrogen hormone molecules by ER-positive breast cancer cells (12).

LHRH agonists desensitise the pituitary gland and downregulate the functions of receptors, thereby suppressing the release of gonadotrophin (13). LHRHs effectuate by ‘turning off’ the production of oestrogen in the ovaries. As a result, fewer oestrogen molecules are available to fuel the growth of ER-positive breast cancer. LHRHs are administered via injection in the stomach once a month or every few months for several months. This form of treatment is common among pre-menopausal women with early-stage ER-positive breast cancer (10).

Fulvestrant belongs to the group of selective ER down-regulators (SERDs), which acts by binding competitively to the ER at a higher affinity than the SERMs. As a pure ER antagonist, Fulvestrant repeals the oestrogen-sensitive gene transcription, ensuring that there is no cross-resistance occurring with other anti-hormone agents. Some preclinical studies showed that Fulvestrant decreased the cellular levels of ER protein and inhibited the ER-induced cell proliferation (1).

Third-generation AIs such as anastrozole, letrozole and exemstane have provided novel approaches to the endocrine hormone therapy of breast cancer. Two types of AIs are steroidal or irreversible (anastrozole and letrozole) and nonsteroidal or reversible (exemestane) inhibitors of oestrogen production. By blocking the aromatase enzyme, AIs suppress the plasma oestrogen levels, reducing the effects of oestrogens in the stimulation of growth in ER-positive breast cancer (1).

### Treatment Resistance

Although treatment with tamoxifen has shown obvious benefits in most ER-positive breast carcinomas that are initially responsive to treatment but may demonstrate reoccurrences, suggesting anti-oestrogen resistance. In Malaysia, approximately 30% of ER-positive tumours are resistant to hormonal therapies or oestrogen deprivation therapies. The patients’ response to endocrine therapy varies between the different ER and PR status (14).

Two types of anti-oestrogen resistance are noted: *de novo* resistance and acquired resistance. *De novo* resistance is encountered among ER-positive breast cancer patients with already a lack of response at the beginning of the treatment. MCF-7 is an example of breast cancer cell line that demonstrates this form of resistance (12). In a previous study, MCF-7 cancer cells transfected with the HER2/Neu oncogene resulted in the induction of tumour growth in xenograft mice despite treatment with tamoxifen (15).

Acquired resistance is shown in ER-positive breast carcinoma that has undergone long term therapy with anti-oestrogen. ER-positive breast tumours, which acquire this form of resistance, continue to exhibit ER expression despite exposure to anti-oestrogen. These cancer cells might operate based on either of the two modes, tamoxifen-non-responsive or tamoxifen-dependent proliferation (12).

In studies conducted by Gottardis and Jordan (16) and O’Regan et al. (17), the acquired resistance was observed when MCF-7 cells were inoculated into ovariectomised athymic mice treated with tamoxifen. A large number of tumours in these mice initially responded to tamoxifen and did not grow; however, some tumours began to grow even in the presence of anti-oestrogen after about a period of a year. Interestingly, the tumours continued to grow in other athymic mice in response to either oestradiol (E2) or tamoxifen.

According to Chang (12), seven different mechanisms underlie the resistance in tamoxifen treatment. Firstly, these mechanisms lack the expression and function of ERα, which leads to the reduced availability of molecular target that ensures the effectiveness of tamoxifen. Secondly, the modified expression of most of the coactivators or coregulators which influence the transcription of ER-mediated genes and the existence of ligand-independent growth factor signalling reactions which induce the activation of kinases and phosphorylation of ER. Thirdly, the modified level of active tamoxifen metabolites is mostly regulated by the drug-metabolising enzymes (DME). Fourthly, the autophagy and apoptosis are inhibited. Interestingly, the presence of ER-negative cancer stem cells can be differentiated from ER-positive cancer cells with inhibited proliferation after exposure to anti-oestrogen therapy. Lastly, cell survival is aided if the repression of antioxidant proteins is avoided by tamoxifen that could eventually backfire, leading to the survival of cancerous cells and the emergence of resistance to tamoxifen-based therapy.

A recent study published by The Institute of Cancer Research (ICR) (18) stated that ER-positive breast cancers produce a molecule made from cholesterol, termed as 25-hydroxycholesterol. It mimics oestrogen and promotes the growth of cancer cells despite the lack of oestrogen during the anti-hormone treatment. These findings were supported by several other studies. The role of HMGCR in premenopausal ER-positive breast cancer patients in response to tamoxifen treatment was predicted (19). The study also revealed that HMGCR was regulated via a negative feedback mechanism in both tamoxifen-sensitive cell line, MCF-7 and tamoxifen-resistant cell line, LCC9 based on the growth requirements and the expression of ER, indicating a positive correlation between the HMGCR activity and ER expression (19).

Based on this evidence, we postulated that cholesterol constituted a novel mechanism of resistance to oestrogen deprivation treatment among ER-positive breast cancer patients.

## Cholesterol Biosynthesis Pathway as a Novel Mechanism of Resistance to Oestrogen Deprivation in ER-Positive Breast Cancer

**Figure 3:**
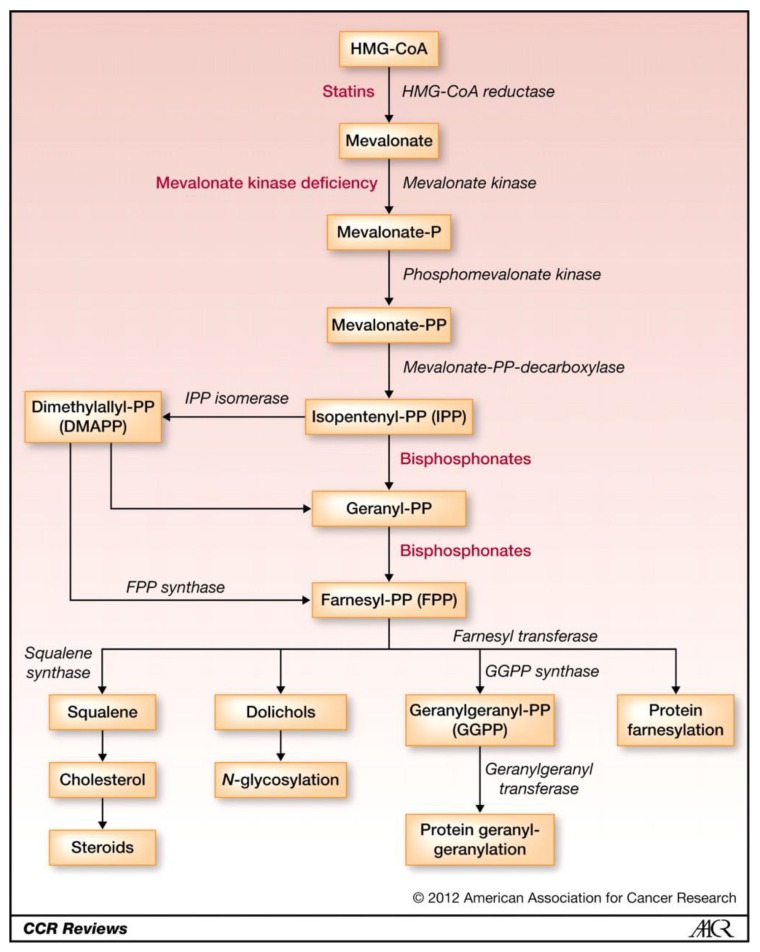
Schematic representation of the mevalonate pathway. The image describes the production of cholesterol and steroids via the activity HMGCR [image taken from Thurnher et al. (20)]

Cholesterol biosynthesis occurs via the mevalonate pathway which is critical for providing the cells with essential bioactive molecules for multiple cellular processes that influence the proliferation of cells. The mevalonate pathway converts mevalonate into producing compounds, such as sterol isoprenoids (cholesterol), steroid hormones and nonsterol isoprenoids (Figure 3). These products play vital roles in various post-translational modifications of several proteins involved in intracellular signalling such as cell proliferation and differentiation (6).

As shown in Figure 3, the earlier step of the mevalonate pathway involves the HMG-CoA conversion of that is catalysed by HMGCR (6). The presence of a low level of sterol isoprenoid (cholesterol) and non-sterol isoprenoids leads to the activation of HMGCR gene transcription by sterol regulatory element binding protein (SREBP). The degradation rate of HMGCR protein depends solely on the requirements of the cells for both sterol isoprenoids (cholesterol) and non-sterol isoprenoids, indicating that the activity of HMGCR is regulated by a negative feedback mechanism via the mevalonate pathway (6).

**Figure 4:**
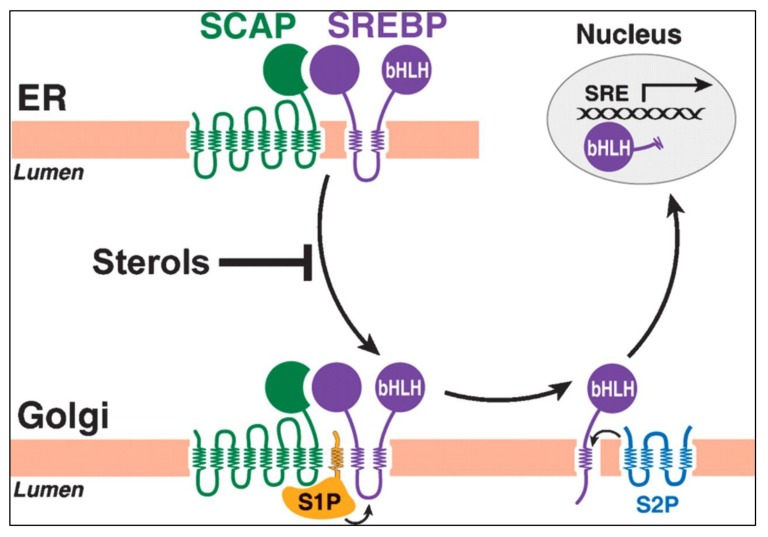
Graphical depiction of SREBP pathway [image taken from Brown and Goldstein (24)]

The Sterol Regulatory Element Binding Protein-2 (SREBP2) is a transcription factor that acts as a sensor for cholesterol production and its activity is negatively regulated by the presence of free cholesterol (21). SREBP2 acts by controlling the expression of numerous genes involved in cholesterol homeostasis, such as HMGCR, FDPS, DHCR7, DHCR24, LSS, FDFT1 and LDLR (22–23). Furthermore, SREBP2 is maintained in an inactive state as a part of a large multi-protein complex associated with the endoplasmic reticulum (24). The integrity of this protein complex is disrupted due to the decreasing level of cholesterol. This causing SREBP2 and its chaperone, SREBP cleavage-activating protein (SCAP) to migrate into the Golgi apparatus and activates a series of proteolytic processing events (Figure 4). Upon entering the nucleus, SREBP2 upregulates the expression of genes, such as HMGCR responsible for the synthesis of cholesterol and those involved in its transport (21).

According to Simigdala et al. (2), the cholesterol biosynthesis pathway was identified as a common adaptive mechanism only in models that have retained the ERs at the point of resistance. In addition, oxysterols such as 25-hydroxycholesterol (25-HC) and 27-hydroxycholesterol (27-HC) increase the transcriptional activity of ER through the recruitment of endogenous oestrogen-regulated genes. Oxysterols are hydroxylated cholesterols with vital roles in cholesterol homeostasis by activating the liver X receptors (LXRs). LXRs are members of a nuclear receptor superfamily of ligand-regulated transcription factors with activity positively regulated by the presence of oxysterol ligands derived from cholesterol found within the cells (21).

ER-positive breast cancer cells become resistant to standard hormone therapy that used cholesterol products such as oxysterols to mimic oestrogen. Recent literature suggested the role of 27-HC as an agonist in ER-positive breast cancer, inducing metastasis and tumour growth despite treatment with anti-hormone therapy. The presence of 27-HC was shown to exert ER agonistic effect. A previous study using murine models demonstrated that the conversion of cholesterol to 27-HC is essential for the proliferation of ER-positive breast cancer cells in an ER-dependent manner (25). This finding shed light on how 27-HC-promoted proliferation of cancer. Also, it serves as the link between hypercholesterolemia and ER-positive breast cancer in postmenopausal women (26).

The study conducted by Simigdala et al. (2) revealed the role of cholesterol biosynthesis as a novel mechanism of resistance to oestrogen deprivation in ER-positive breast cancer. The study concluded that the cholesterol biosynthesis was a commonly upregulated pathway in the ER + LTED but not the ER-LTED cell lines, suggesting a potential mechanism dependent on the continued expression of ER. Strikingly, 25-HC and 27-HC showed selective oestrogen molecule modulator activity and rescued the anti-proliferative effects of Fulvestrant. Based on this result, the study proposed that ER-positive LTED cells synthesised increased levels of 25-HC and 27-HC, leading to increased ER activity in response to the loss of E2 proliferation. Also, increased expression of enzymes, involved in cholesterol biosynthesis, such as MSMO1, EBP, LBR and SQLE were used in *in silico* analysis of two independent studies on primary ER-positive breast cancer patients treated with anti-hormone therapy.

**Figure 5:**
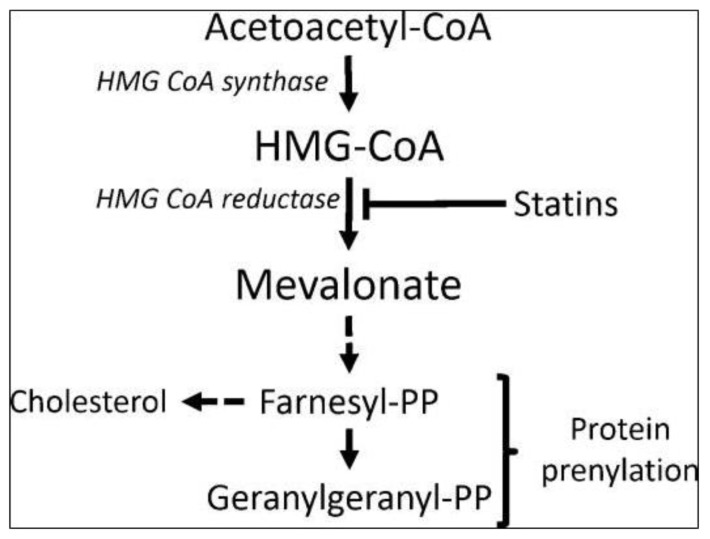
Effect of statin on the mevalonate pathway. Statin interrupts the enzymatic reduction of HMG-CoA to mevalonate by inhibiting the activity of HMGCR. The interruption inhibits the synthesis of cholesterol and isoprenoids such as farnesyl-PP and geranylgeranyl-PP [image taken from Ahern et al. (29)] Note: The dashed arrows represent the multi-step transitions in the mevalonate pathway

Several studies used statin as part of ER-positive breast cancer treatment (27–28). Statin is a lipid-lowering drug commonly used to treat hyperlipidaemia and targeted the mevalonate pathway. Statins act by inhibiting the HMGCR enzyme (Figure 5). However, the relevance of statins in the reduction of the risk of cancer is yet controversial (30). The effect of statin inhibition on HMGCR is often associated with the characteristics, prognosis and treatment response of breast tumour (26).

Thus, instead of using statin as a part of the ER-positive treatment, we would like to propose the use of andrographolide, a natural product to target the cholesterol biosynthesis pathway to manage the occurrence of breast cancer resistance to hormone therapy. Since the chemical structure of andrographolide and statins are similar (4), we speculated that andrographolide could serve as an alternative owing to its natural origin. Thus, taken together, it can be concluded that natural products can target specific sites to prevent the proliferation of tumour cells, minimising the side effects.

## Introduction to Andrographolide as an Alternative for Breast Cancer Treatment

### Introduction to Andrographolide

Andrographolide, a diterpene lactone, is the major phytoconstituent or the bioactive component extracted from *Andrographis paniculata* Nees (31). This plant is commonly found in Asian countries such as India, Thailand, Indonesia and throughout Southeast Asia. *Andrographis paniculata* belongs under the *Acanthaceae* family along with *Clinacanthus nutans. Andrographis paniculata*, also known as ‘the King of Bitters’ has been traditionally used to treat ailments such as respiratory infection, bacterial dysentery and excessive diarrhoea. It is also known as ‘Hempedu Bumi’. Reportedly, this plant possesses hypolipidemic effect (32), anti-inflammatory (33), anti-cancer (34), anti-diabetic (35), anti-oxidant (36), anti-malarial (37) and anti-HIV properties (38).

**Figure 6:**
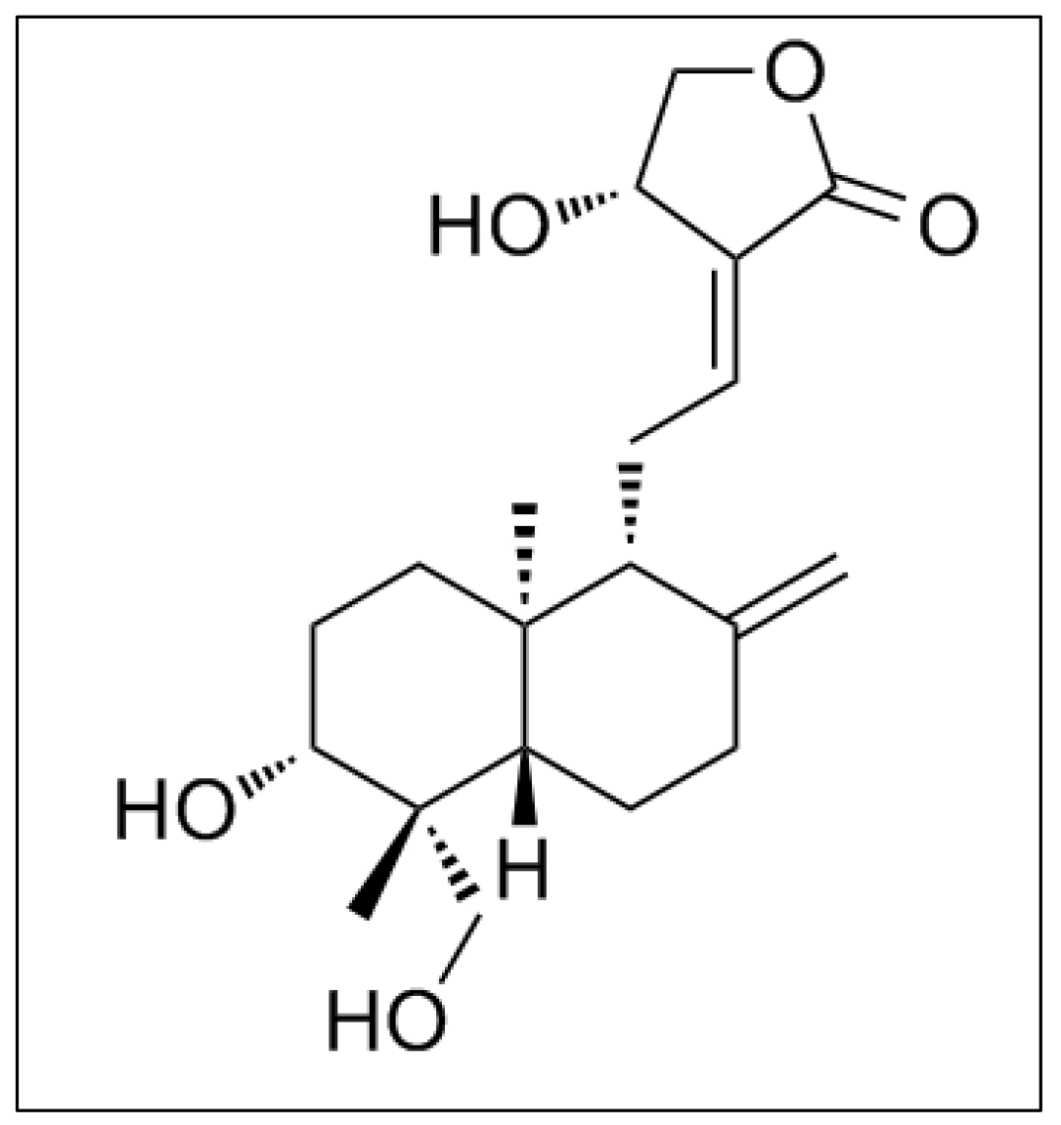
Chemical structure of andrographolide [image adapted from Jada et al. (39)]

The basic structural skeleton of andrographolide is composed of three hydroxyl groups at C3 (secondary), C14 (allylic) and C19 (primary) (Figure 6) (39). Moreover, the chemical structure of andrographolide is responsible for its biological and medicinal properties described previously (39).

### Inhibitory Action of Andrographolide in Cancer

**Figure 7:**
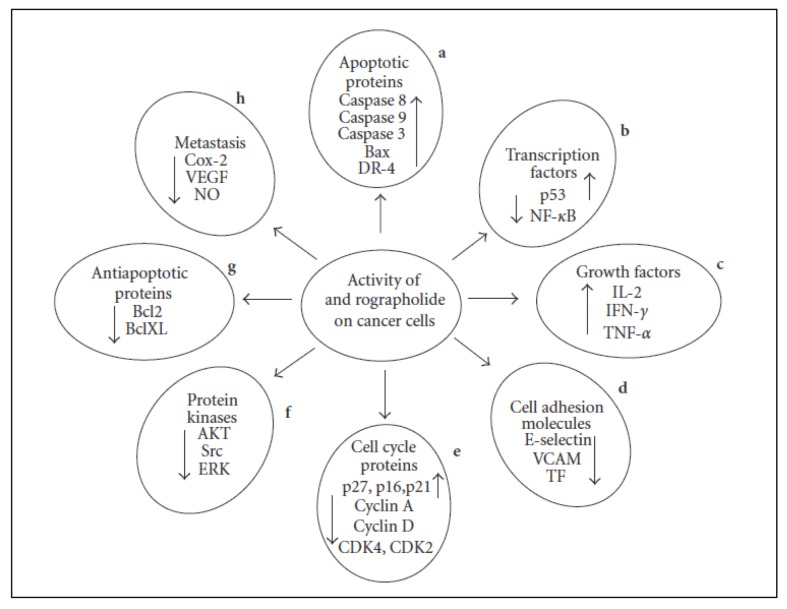
Effects of andrographolide treatment on cancer cells. Factors required for tumour progression, nourishment and metastasis such as cyclins A, D, Cdk2, Cdk4, NF-κB, VEGF, E-selectin, VCAM, Akt, TNF and Bcl2 were downregulated. On the other hand, tumour suppressor elements such as p53, caspases and inhibitory proteins p21, p16 and p27, were upregulated as observed in various studies investigating the anti-cancer potential of andrographolide [image taken from Varma et al. (40)]

Andrographolide inhibits the growth of cancer cells via several mechanisms, such as cytotoxic activity, induction of cell cycle arrest, induction of apoptosis, immunomodulatory effect, anti-inflammatory and anti-angiogenic activities and chemoprotective mechanism (40) (Figure 7).

In a study conducted by Harjutaruno et al. (41), andrographolide was tested on TD-47 ER-positive breast cancer cell line. Subsequently, the study demonstrated that andrographolide exerts anticancer properties by inducing apoptosis via increased expression of p53 (a tumour suppressor protein), Bax protein and caspase-3. In addition, andrographolide also decreased the expression of Bcl-2 as determined by immunohistochemistry analysis.

In another study, andrographolide was shown to possess anti-cancer properties based on its ability to block cell cycle proliferation at the G_0_ and G_1_ phases in ER-positive breast cancer cells by disrupting the growth-related signalling pathway (42). This finding was further supported by Banerjee et al. (43), the study which stated that andrographolide treatment on ER-positive breast cancer cells enhances the production of reactive oxygen species (ROS), contributing to the loss of matrix metalloproteinases (MMPs), activating caspase-9 and -7 and externalising phosphatidyl serine. These modifications to the proteins that are part of the growth signalling pathways allow the inhibition of cell proliferation of ER-positive breast cancer cells.

The study by Liang et al. (44) demonstrated that andrographolide possesses anti-tumour properties by targeting the level of oncoprotein, known as v-Src, via attenuation of Erk1/2 signalling pathway. In terms of the expression of cyclin-dependent kinases (CDKs), andrographolide downregulates the expression of CDK-4, which indirectly causes arrest in the G1 phase of the cell cycle (39). Also, andrographolide suppresses the rate of cell proliferation in ER-positive breast cancer cells via the induction of p27 (45), which is supported by a study by Jada et al. (39). Moreover, andrographolide inhibits 12-O-(TPA) induced by ER-positive breast cancer cell invasion. This suppression was potentially associated with the upregulated level of heme oxygenase and the downregulated expression of MMP9 (46).

The suppression of the majority of the growth signalling pathways described above eventually leads to the suppression of NF-κB, which acts as the main switch in the proliferation of ER-positive breast cancer cells. Therefore, by ‘turning off’ this main switch, the proliferation of ER-positive breast cancer cells can be inhibited, thereby preventing the development of breast carcinoma (4, 46–48).

A recent study by Kang et al. (49) discovered that nanomedicine-based combination therapy involving doxorubicin and andrographolide inhibited the growth of tumour in orthotopic breast tumour mouse model. Additionally, andrographolide is also used to treat other types of cancers such as colon cancer (50–51), lymphoma (52), lung cancer (53) and pancreatic cancer (54). In a study by Zhang et al. (50), andrographolide suppressed cell proliferation by inducing cytotoxicity, evoking cell apoptosis and activating caspase-3/9 activities in human colon cancer cells, SW620. Furthermore, the results revealed that the anti-proliferation effects of andrographolide on SW620 cells were associated with the inhibition of activated TLR4, MyD88, NF-κB-p65 and MMP-9 signalling activation.

In another study conducted using gastric cells, andrographolide inhibited the cell proliferation, invasion, migration and cell cycle arrest and promoted apoptosis in SGC7901 cells. The underlying mechanisms showed an upregulation of Timp-1/2, cyclin B1, p-Cdc2, Bax and Bik expression and the downregulation of MMP-2/9 and anti-apoptosis protein Bcl-2 expression (55). In prostate cancer, andrographolide inhibited the growth of cancer cells by targeting the cell cycle regulators: CXCR3 and CXCR7 chemokine receptors (56).

### Andrographolide is a Lipid-Lowering Agent and Its Hypolipidemic Mechanisms

According to several studies, andrographolide could act as a lipid-lowering as well as a hypolipidemic agent. This postulate has been proved by various study designs and methods.

A study by Nugroho et al. (57) found that the purified extract of *Andrographis paniculata* containing andrographolide exhibited anti-hyperlipidaemic effects in high fructose fatfed rats. Consequently, the purified extract containing andrographolide significantly reduced the levels of cholesterol, triglyceride (TG) and low-density lipoprotein (LDL) in the treated model as compared to the control.

The anti-hyperlipidaemic effect of andrographolide was highlighted in a similar study conducted on C57BL/6 mice fed high-fat diet (HFD) to induce obesity. In this study, we found that andrographolide downregulated the expression of human SREBP target genes and decreased the cellular lipid accumulation in vitro. Furthermore, the study also noted that andrographolide attenuated HFD-induced body weight gain and fat accumulation in the liver or adipose tissues, thereby improving the levels of serum lipid levels in the HFD-induced obese mice (5). This study further explained the hypolipidemic property of andrographolide by investigating the effects of andrographolide on SREBP activity and genes involved in the fatty acid and TG synthetic pathways such as SREBP-1, FAS and acetyl-coenzyme A carboxylase alpha (ACC-1). In addition, the study also focuses on the genes involved in cholesterol synthesis such as SREBP-2, HMGCR and lanosterol synthase (LSS). In addition, andrographolide downregulated the expression of SREBP target genes and decreased the cellular lipid content. Strikingly, 10 μM andrographolide inhibited the trans-activity of SREBP by 68.7%, indicating that andrographolide might counteract the activity of SREBP. Also, andrographolide significantly downregulated the genes involved in the fatty acid and TG pathway and cholesterol synthesis, although the target genes of LXRs were not affected (5).

In a study by Batran et al. (58), the hypolipidemic effect of andrographolide was investigated on hyperlipidaemia induced by *Porphyromonas gingivalis* in rats. The study found that the total serum cholesterol (TC), LDL-C and TG levels of andrographolide-treated group were significantly reduced as compared to untreated group. In addition, the liver tissues of the groups treated with andrographolide exhibited a reduced accumulation of lipid droplets. In terms of lipid peroxidation, the study found that malondialdehyde (MDA) (biomarker for oxidative stress) level was low in treated groups, while that of antioxidant enzymes, superoxide dismutase (SOD) and glutathione peroxidase (GPx) were increased significantly. The present study not only highlights the hypolipidemic effect of andrographolide but also reflects the potent antioxidant properties of the drug (58).

An advanced technology study using nanoparticles was conducted to investigate the development, characterisation and toxicity of nanoparticles containing andrographolide. The total cholesterol and TG levels were found to be downregulated in the treatment group as compared to the control group (59).

**Figure 8:**
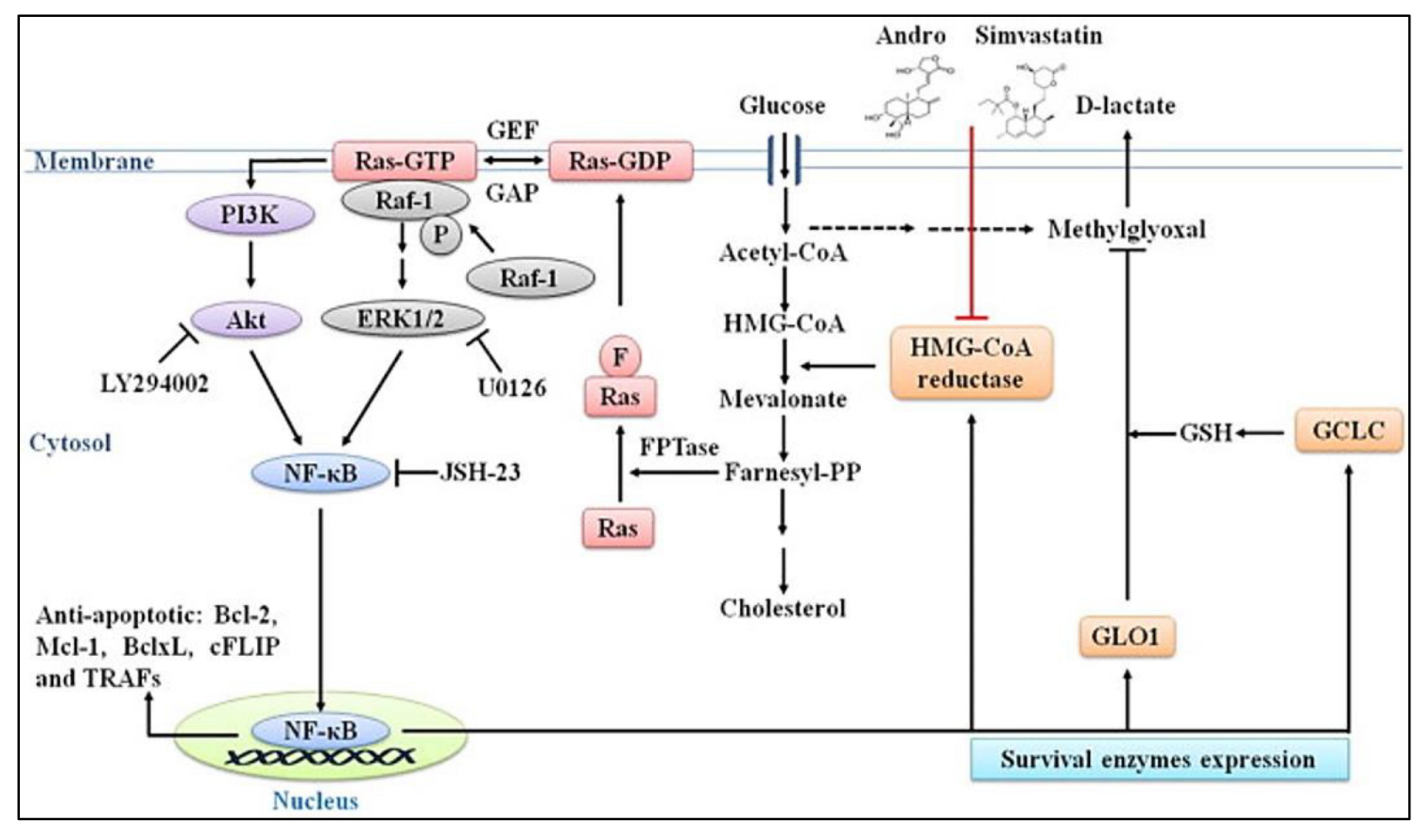
Effect of andrographolide and Simvastatin on the mevalonate pathway in HL-60 leukaemia cells. Andrographolide inhibits the HMGCR activity and this affects the downstream pathway of cholesterol biosynthesis [image taken from Chen et al. (4)]

Previously, andrographolide treatment was shown to reduce the cholesterol level and inhibit the activity of HMGCR in HL-60 leukaemia cells, which was similar to the effects of Simvastatin treatment (Figure 8) (4). These effects were further associated with the downregulation of Ras protein, the subsequent inactivation of the downstream phosphorylation of Akt and ERK and the blockage of NF-κB translocation.

## Conclusion

To date, no study has been conducted to elucidate the hypolipidaemic effects of andrographolide in preventing the growth of ER-positive breast cancer and the occurrence of resistance via cholesterol biosynthesis. Thus, this review opened up new perspective of ideas for the use of natural resources to treat ER-positive breast cancer and prevent treatment resistance. Further studies are needed to investigate the potential of andrographolide in blocking the cholesterol biosynthesis pathway as a novel mechanism to counter treatment resistance. Therefore, future studies should focus on the effects of andrographolide on the downstream pathway of cholesterol biosynthesis in ER-positive breast carcinoma and the production of oxysterols such as 25-HC and 27-HC.
